# Supplementary material for: Cancer risk perception and physician communication behaviors on cervical cancer and colorectal cancer screening
Source: eLife. 2021 Aug 24;10:e70003. doi: 10.7554/eLife.70003 (PMC8384416; doi:10.7554/eLife.70003)
Supplement: Supplementary file 1. [file elife-70003-supp1.docx]

**Supplemental Table 1**. Screening outcomes by total population religion and by MENA ethnicity

|  |  | **Both Cervical and CRC Screens** | | **Cervical cancer alone** | | **CRC alone** | | **Neither screen** | |
| --- | --- | --- | --- | --- | --- | --- | --- | --- | --- |
|  |  | **N** | **Row%** | **N** | **Row%** | **N** | **Row%** | **N** | **Row%** |
| **Total population*** |  |  |  |  |  |  |  |  |  |
| Christian ƚ |  | 141 | 59 | 41 | 17 | 27 | 11 | 31 | 13 |
| Muslim |  | 7 | 27 | 13 | 50 | 1 | 4 | 5 | 19 |
| Jewish |  | 10 | 72 | 2 | 14 | 2 | 14 | 0 | 0 |
| No Religion/No god |  | 50 | 52 | 22 | 22 | 13 | 13 | 13 | 13 |
|  |  |  |  |  |  |  |  |  |  |
| **MENA population** |  |  |  |  |  |  |  |  |  |
| Christian ƚ |  | 2 | 17 | 6 | 50 | 1 | 8 | 3 | 25 |
| Muslim |  | 6 | 25 | 13 | 52 | 1 | 4 | 5 | 20 |

Ƚ Christian includes Chaldean, Coptic, Assyrian, Catholic, Jehovah Witness

*H(3,N=378)=7.8, p<0.05 where Muslim women screen differently than Christian (p<0.05) and differently from those women with no religion or no belief in a god (p<0.05). Among MENA women, religion was not associated with screening behavior.
