# Supplementary material for: Cancer risk perception and physician communication behaviors on cervical cancer and colorectal cancer screening
Source: eLife. 2021 Aug 24;10:e70003. doi: 10.7554/eLife.70003 (PMC8384416; doi:10.7554/eLife.70003)
Supplement: Supplementary file 2. [file elife-70003-supp2.docx]

**Supplemental Table 2**. Agreement with cancer risk perceptions and physician communication behaviors by self-declared religion

|  | **Christian** | | **Muslim** | | **Jewish** | | **No Religion/**  **No god** | |
| --- | --- | --- | --- | --- | --- | --- | --- | --- |
|  | **N** | **%** | **N** | **%** | **N** | **%** | **N** | **%** |
| There’s not much you can do to lower your chances of getting cancer. ǂ | 194 | 83 | 17 | 65 | 13 | 93 | 74 | 76 |
| When I think about cancer, I automatically think about death. § | 115 | 49 | 4 | 15 | 9 | 64 | 43 | 44 |
| Involve you in decisions about your health care as much as you wanted.* | 197 | 89 | 17 | 71 | 12 | 86 | 73 | 85 |

ǂ H (1, N=360)=8.5, p<0.01

§ H(1, N=361)=3.9 p<0.05

* H(1, N=334)=13.7, p<0.001

Muslim women have the lowest agreement among all religions for each of the cancer risk perceptions and physician communication behaviors.
