# Supplementary material for: Cancer risk perception and physician communication behaviors on cervical cancer and colorectal cancer screening
Source: eLife. 2021 Aug 24;10:e70003. doi: 10.7554/eLife.70003 (PMC8384416; doi:10.7554/eLife.70003)
Supplement: Supplementary file 3. [file elife-70003-supp3.docx]

Supplemental Table 3. Cancer screenings by religion and race/ethnicity

|  | **BOTH screens compared to NEITHER** | | | **CRC alone compared to NEITHER** | | | **BOTH compared to CRC alone** | | | **CRC only compared to Cervix alone** | | | **BOTH compared to Cervix alone** | | |
| --- | --- | --- | --- | --- | --- | --- | --- | --- | --- | --- | --- | --- | --- | --- | --- |
|  | **aOR** | **L95** | **U95** | **aOR** | **L95** | **U95** | **aOR** | **L95** | **U95** | **aOR** | **L95** | **U95** | **aOR** | **L95** | **U95** |
| **Religion** |  |  |  |  |  |  |  |  |  |  |  |  |  |  |  |
| Christian | referent |  |  | referent |  |  | referent |  |  | referent |  |  | referent |  |  |
| Muslim | 1.82 | 0.45 | 7.40 | 0.69 | 0.09 | 5.23 | 1.46 | 0.19 | 11.13 | 0.64 | 0.09 | 4.31 | 1.21 | 67972.95 | 1.89 |
| No Religion/ No god | 0.66 | 0.28 | 1.55 | 1.22 | 0.37 | 4.01 | 0.82 | 0.25 | 2.69 | 1.07 | 0.35 | 3.24 | 0.58 | 0.28 | 1.21 |
| **Race/Ethnicity** |  |  |  |  |  |  |  |  |  |  |  |  |  |  |  |
| White | referent |  |  | referent |  |  | referent |  |  | referent |  |  | referent |  |  |
| Black | 2.09 | 0.95 | 4.62 | 1.17 | 0.42 | 3.27 | 0.86 | 0.31 | 2.39 | 1.37 | 0.52 | 3.59 | **2.44** | **1.21** | **4.95** |
| MENA | **0.24** | **0.07** | **0.80** | 0.53 | 0.12 | 2.42 | 1.88 | 0.41 | 8.58 | 0.39 | 0.09 | 1.62 | **0.17** | **0.06** | **0.52** |

Bold/red is significant

Adjusted for religion and race

MENA women, not Muslim women, were more likely to have neither screen than dual screening and more likely to have cervical cancer screening than dual screening. Black women were more likely to have dual screening than cervical cancer screening alone.
